# Genetic engineering of *Nannochloropsis oceanica* to produce canthaxanthin and ketocarotenoids

**DOI:** 10.1186/s12934-024-02599-4

**Published:** 2024-11-29

**Authors:** Davide Canini, Flavio Martini, Stefano Cazzaniga, Tea Miotti, Beatrice Pacenza, Sarah D’Adamo, Matteo Ballottari

**Affiliations:** 1 Dipartimento di Biotecnologie, Università Degli Studi di Verona, Strada le Grazie 15, 37134 Verona, Italy; 2 Bioprocess Engineering Chair Group, Wageningen University and Research, Wageningen, 6700 AA The Netherlands

**Keywords:** Ketocarotenoids, Microalgae, Canthaxanthin, Carotenoids, *Nannochloropsis*

## Abstract

**Background:**

Canthaxanthin is a ketocarotenoid with high antioxidant activity, and it is primarily produced by microalgae, among which *Nannochloropsis oceanica*, a marine alga widely used for aquaculture. In the last decade, *N. oceanica* has become a model organism for oleaginous microalgae to develop sustainable processes to produce biomolecules of interest by exploiting its photosynthetic activity and carbon assimilation properties. *N. oceanica* can accumulate lipids up to 70% of total dry weight and contains the omega-3 fatty acid eicosapentaenoic acid (EPA) required for both food and feed applications. The genome sequence, other omics data, and synthetic biology tools are available for this species, including an engineered strain called LP-tdTomato, which allows homologous recombination to insert the heterologous genes in a highly transcribed locus in the nucleolus region. Here, *N. oceanica* was engineered to induce high ketocarotenoid and canthaxanthin production.

**Results:**

We used *N. oceanica* LP-tdTomato strain as a background to express the key enzyme for ketocarotenoid production, a β-carotene ketolase (CrBKT) from *Chlamydomonas reinhardtii*. Through the LP-tdTomato strain, the transgene insertion by homologous recombination in a highly transcribed genomic locus can be screened by negative fluorescence. The overexpression of CrBKT in *bkt* transformants increased the content of carotenoids and ketocarotenoids per cell, respectively, 1.5 and 10-fold, inducing an orange/red color in the *bkt* cell cultures. Background (*LP*) and *bkt* lines productivity were compared at different light intensities from 150 to 1200 µmol m^-2^ s^-1^: at lower irradiances, the growth kinetics of *bkt* lines were slower compared to *LP*, while higher productivity was measured for *bkt* lines at 1200 µmol m^-2^ s^-1^. Despite these results, the highest canthaxanthin and ketocarotenoids productivity were obtained upon cultivation at 150 µmol m^-2^ s^-1^.

**Conclusions:**

Through targeted gene redesign and heterologous transformation, ketocarotenoids and canthaxanthin content were significantly increased, achieving 0.3% and 0.2% dry weight. Canthaxanthin could be produced using CO_2_ as the only carbon source at 1.5 mg/L titer. These bkt-engineered lines hold potential for industrial applications in fish or poultry feed sectors, where canthaxanthin and ketocarotenoids are required as pigmentation agents.

**Supplementary Information:**

The online version contains supplementary material available at 10.1186/s12934-024-02599-4.

## Background

Microalgae are interesting microbial cell factories due to their ability to exploit light energy to fix CO_2_ into organic compounds with different industrial applications [1–8]. Microalgae have several advantages over land plants, mainly because these microorganisms can be cultivated under controlled conditions (nutrients, CO_2_, O_2_, light, pH) in photobioreactors and on non-arable land, avoiding competition with food crops. On the other hand, a few problems must be faced, such as the risk of contamination, water consumption, and downstream costs [9, 10].

*Nannochloropsis oceanica* is a microalgal species from the superphylum Heterokonta (or Stramenopiles) that offers potential solutions to some of these problems. Indeed, it grows in seawater, reducing the risk of contamination and freshwater consumption, and accumulates high lipid content, including the high-value omega-3 fatty acid eicosapentaenoic acid (EPA) that may compensate for the elevated downstream costs [11–14]. For this reason, *Nannochloropsis spp.* is industrially established as a feed ingredient with a current market size of 4 million dollars with a projection to 15 million dollars in 2028–2032 (https://www.gminsights.com/industry-analysis/nannochloropsis-market).

During the last decade, *N. oceanica* has emerged as a model organism for oleaginous microalgae: it has a small haploid nuclear genome, with a low degree of functional redundancy and low intron-density compared to the model chlorophyte *Chlamydomonas reinhardtii* [15]. A successful transformation protocol by homologous recombination has already been demonstrated in *Nannochloropsis spp.*, allowing straightforward genetic engineering strategies [16]. More recently, efficient transgene expression was achieved by exploiting a highly transcribed genomic locus: A background strain called LP-tdTomato (LP) was developed carrying a fluorescent protein-encoding gene (tdTomato), which can be substituted by homologous recombination with the heterologous gene of interest, allowing high -throughput selection of transformant lines by negative fluorescence screening [17]. Moreover, a dedicated system and synthetic biology database, NanDeSyn, is available for *N. oceanica* [18].

**Fig. 1:**
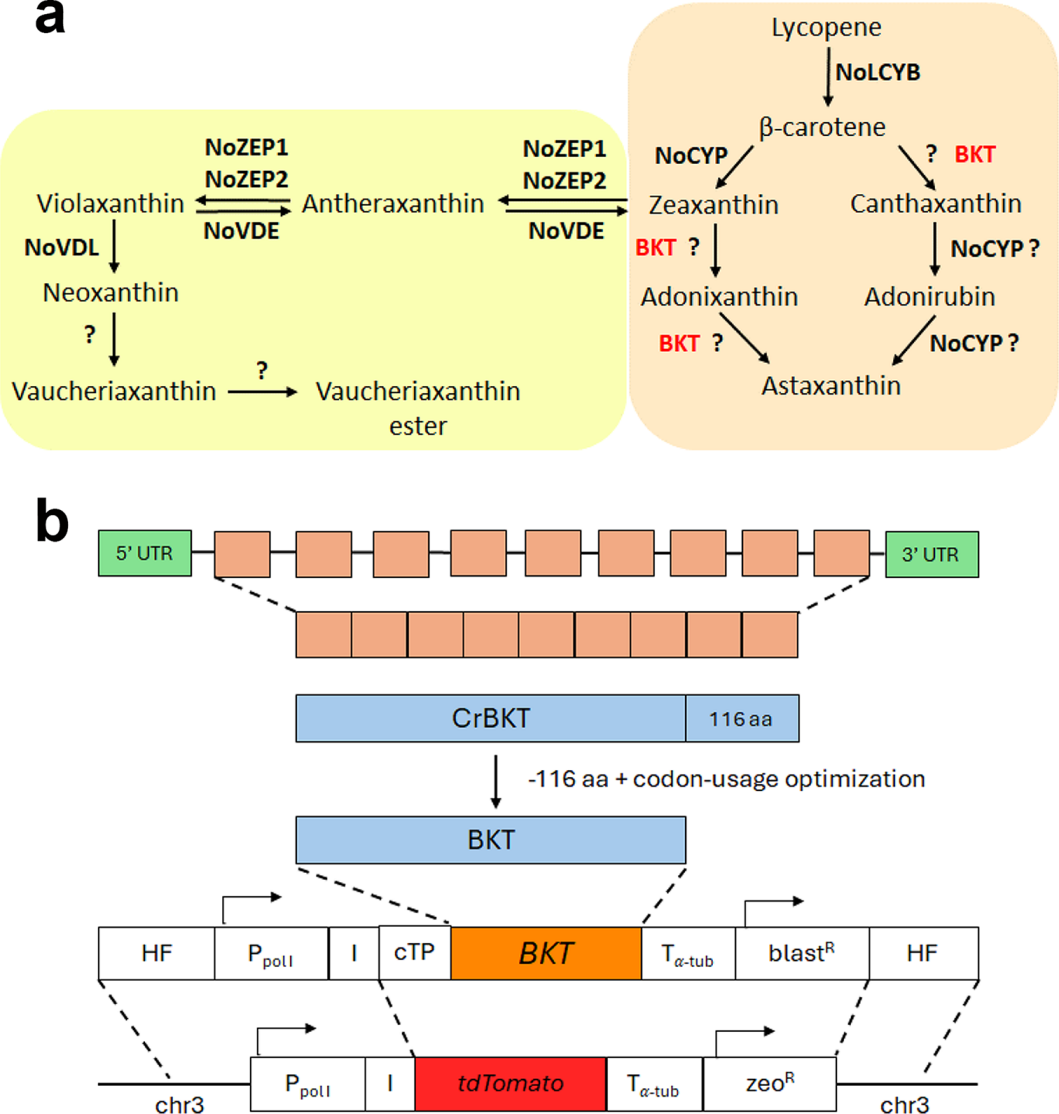
CrBKT expression in *Nannochloropsis oceanica.* (**a**) Schematic representation of the carotenoid pathway towards astaxanthin biosynthesis (**b**) Schematic diagram of the expression vector used to transform *N. oceanica LP* background strain

Carotenoids are among the compounds produced by microalgae, generating significant interest for industrial applications in different sectors, such as feed, food, and cosmetics [19–22]. Astaxanthin is well known for its potent antioxidant properties, and it is considered one of the primary bioproducts on the market produced from microalgae, mainly from the green alga *Haematococcus pluvialis*, also known as *H. lacustris*. Another ketocarotenoid with an important industrial application is canthaxanthin, a precursor in the astaxanthin biosynthesis: canthaxanthin is, indeed, produced by the ketolation of beta-carotene catalyzed by the enzyme beta-carotene ketolase (BKT) [23]. A subsequent hydroxylation reaction converts canthaxanthin into astaxanthin (Fig. 1a). The antioxidant properties of astaxanthin and canthaxanthin are similar [24]. Still, while both are commonly used in aquaculture as pigmentation agents [25, 26], the latter has specific applications mainly as an additive in the feed of laying hens to improve yolks’ pigmentation [27]. The global market of canthaxanthin is currently valued at around 250 million €. Moreover, a positive effect on human health has been reported for canthaxanthin, which enriches LDL and may potentially protect cholesterol from oxidation [28]. In addition, the canthaxanthin immunomodulatory activity and its role in gap junction communication have been reported [28]. Canthaxanthin can be found in nature in mushrooms such as golden chanterelle mushrooms which are rich in canthaxanthin, as well as vegetables like yellow bell peppers and corn, and in several microalgae, such as Dunaliella, although in low amounts. Despite the industrial interest in canthaxanthin, few research efforts were conducted to improve its production in photoautotrophic organisms. Metabolic engineering of *Dunaliella salina* by the heterologous expression of BKT enzyme from *H. pluvialis* has led to canthaxanthin accumulation of up to 0.19% of dry weight under stress conditions [29]. Slightly lower values (~ 0.16%) were obtained in the case of *Synechococcus* using a similar metabolic engineering approach [23]. In the case of *Chlamydomonas reinhardtii*, a BKT enzyme (CrBKT) is present in its nuclear genome; however, the gene is poorly expressed [30], and the microalga does not accumulate ketocarotenoids under any condition. A comparison with known BKT protein sequences showed that CrBKT has a longer C-terminal tail when compared to active homologous. The synthetic redesign and the expression of the optimized truncated CrBKT, which removes the extra residues at the C-terminus allowed the conversion of 70% of carotenoids into ketocarotenoids in *C. reinhardtii* most of which were astaxanthin [30]. Carotenoid biosynthesis in *N. oceanica* relies on an isopentyl phosphate pool produced by the 2-C-methylerythritol 4-phosphate (MEP) pathway [31]. *N. oceanica* has a phytoene desaturase (NoPDS), a lycopene β-cyclase (NoLCYB) a cytochrome P450-dependent β-carotene hydroxylase (NoCYP97F5), two zeaxanthin epoxidases (NoZEP1, NoZEP2), a violaxanthin de-epoxidase (NoVDE) and a violaxanthin de-epoxidase-like (VDL) [32–34]. Although the low levels of the ketocarotenoids canthaxanthin and astaxanthin suggest the activity of a β-carotene ketolase [35], bioinformatics analyses from several research groups have not identified any gene likely encoding this enzyme [20, 32]. One approach to increasing canthaxanthin and other ketocarotenoids content is the generation of novel genotypes by chemical mutagenesis (UV) and screening for higher-accumulating mutants. This strategy was successfully applied in *N. gaditana*, resulting in a mutant named S4, which exhibited increased ketocarotenoid productivity [20]. However, S4 mutant accumulated canthaxanthin only up to ~ 15% of the total carotenoids. To improve ketocarotenoids and canthaxanthin accumulation, this work employed a synthetic biology approach by redesigning the BKT encoding gene from *Chlamydomonas reinhardtii* (*CrBKT)* and introducing it into *N. oceanica* by homologous recombination at a highly transcribed locus previously identified in the LP-tdTomato background strain.

## Methods

### Strains and growth conditions

*N. oceanica* strains IMET1 (WT) and LP-tdTomato (LP) were previously described [17]. Artificial Seawater Medium (ASW) was used as a growth medium. 1.5% agar was added to ASW for cultivation in a solid medium. *N. oceanica* cultivation was performed in flasks with an initial concentration of 5 × 10^6^ cells/mL, under continuous light at 40 µmol m^− 2^ s^− 1^ intensity, with atmospheric CO_2_ and orbital shaking at 150 rpm.

### DNA construct design

To obtain a transformant strain of *N. oceanica* with increased content of ketocarotenoids, we designed a construct for the overexpression of a β-carotene ketolase from *C. reinhardtii* (CrBKT) [30]. *N. oceanica* LP-tdTomato (LP) strain was used as background for inserting heterologous genes through homologous recombination in a highly expressed locus in chromosome 3, facilitating negative-fluorescent screening [17]. The *CrBKT* gene was optimized by removing 116 amino acids from the Cre04.g215000 protein sequence of *C. reinhardtii*, as reported by [30], resulting in a remaining 328 amino acids that were translated into a codon-optimized CDS based on *N. oceanica* codon usage by OPTIMIZER software [35]. The optimized *CrBKT* gene sequence for *N. oceanica* transformation is provided in Additional File 1. The BKT construct for homologous recombination includes the codon-optimized *CrBKT* gene for *N. oceanica*, a chloroplast target peptide (cTP) from *N. oceanica* VCP1 gene, a transcriptional terminator from the alpha-tubulin (T_α−tub_) gene from *N. oceanica*, Blasticidin S resistance cassette driven by the transcriptional promoter of lipid droplet surface protein (LDSP) gene from *N. oceanica*, Blasticidin-S deaminase from *Aspergillus terreus* and a transcriptional terminator from Cauliflower Mosaic Virus (CaMV) 35 S. Additionally, it contains the transcriptional promoter of Polymerase I (P_PolI_) from *N. oceanica*, partial ITS2 + 25 S rDNA from *N. oceanica*, *N. oceanica* IRES element, and two homologous flanks (HF).

### Plasmid construction

The plasmid pCpTE-pPolI was used as the backbone [17]. *CrBKT* optimized gene was ordered by Eurofins Genomics. Both pCpTE-pPolI and *CrBKT* were amplified by Phusion™ High-Fidelity DNA Polymerase (ThermoFisher) and subsequently assembled using Gibson Assembly^®^ Master Mix – Assembly (E2611) (New England Biolabs) according to manufacturer instructions, with 25 nucleotide overlap. The plasmid sequence of pCpTE-pPolI-BKT was confirmed by sequencing, and the BKT construct was amplified by Phusion™ High-Fidelity DNA Polymerase (ThermoFisher). The primers used for amplification reactions required for Plasmid construction are listed in Additional File 1.

### Nannochloropsis Oceanica transformation

*N. oceanica LP* strain was transformed using electroporation, as described in references [17] and [36]. *LP* strain was cultivated in a flask under 80 µmol m-2s-1 continuous light, and 50 mL was collected during the exponential growth phase by centrifugation (2500×g, 5 min, 4 °C). Cell pellets were washed thrice with cold 375 mM sorbitol and resuspended in 200 µL (around 2.5 × 10^9^ cells ml^− 1^). The cell suspension was mixed with 1 µg of construct DNA in pre-cooled 2 mm electroporation cuvettes and rapidly pulsed using a Bio-Rad GenePulser II, set to exponential pulse decay with 2.2 kV electric field strength, 50 µF capacitance, and 600 Ω resistance. After the ~ 25 ms pulse, cells were immediately transferred to 5 ml ASW-NB and recovered for 24 h under 40 µmol m^− 2^s^− 1^ without agitation. Subsequently, cells were pelleted (2500×g, 10 min), resuspended in a small amount of supernatant, plated onto ASW plates containing 100 µg ml-1 Blasticidin S, and incubated for three weeks during the recovery period.

### Transformant screening and confirmation

After 3 weeks of transformation, selected resistant colonies were transferred to a new ASW plate to maintain the selective pressure. After one week, cells were inoculated in 24well plates and incubated under 40 µmol m^− 2^s^− 1^ continuous light and orbital shaking around 150 rpm. After three days, the transformants were analyzed for tdTomato fluorescence by a plate reader (Tecan Infinite^®^ 200) with excitation at 523 nm and emission at 627 nm. Colonies exhibiting an essentially absent tdTomato fluorescence signal (similar to the WT case) were selected, and PCR confirmed the correct DNA insertion. The primers used for this process are listed in Additional File 1.

### Cultivation in photobioreactor

To compare *LP* and *bkt* strains (*bkt2*, *bkt3*), they were cultivated in a controlled 80 mL airlifted photobioreactor (Multi-Cultivator MC 1000-OD, Photon Systems instruments™). First, cells with an initial concentration of 2 × 10^7^ cells mL-1 were grown at 25 °C, 3% CO_2_ enriched air insufflation, and continuous light at 150 µmol m-2 s-1 for two days, then the light intensity was set to the desired intensity (150, 600, or 1200 µmol m^− 2^ s^− 1^) for 2 additional days. After the adaptation, cultures were refreshed with an initial concentration of 5 × 10^6^ cells mL^− 1^ at 25 °C, 3% CO_2_ enriched air insufflation, and grown with continuous light at 150, 600, or 1200 µmol m^− 2^ s^− 1^. To determine the effect of nitrogen repletion, depletion, and extremely high light (3000 µmol m^− 2^s^− 1^) on *bkt* strains, cultures grown under 150 µmol m^− 2^s^− 1^ were centrifuged (3300 xg, 10 m, RT) in stationary phase, resuspended by fresh ASW or ASW with no nitrogen. The cultures were cultivated in these conditions for 3 days. To determine the biomass dry weight (DW), at the end of the growth, a 50 mL culture sample was collected and centrifuged (3300 xg, 10 m, RT), the supernatant was discarded, the pellet sample lyophilized for 48 h, and then weighed.

### Biomass and carotenoids productivity determination

Average volumetric biomass productivity at the cultivation time t (P_t.ave_) was calculated considering the final dry weight concentration of the biomass harvested at time t as described in the following equation.

$$\:{\text{P}}_{\text{a}\text{v}\text{e}}=\:\frac{\text{c}}{\text{t}}$$

c: final biomass DW concentration (g L^− 1^); t: cultivation time (days).

Maximal volumetric biomass productivities (P_max_) at the cultivation time t were calculated upon fitting of growth curves (OD_720_) by the sigmoidal Boltzman model function. The fitted curves were then used to calculate the 1st derivative function. The maximum value and the median value of the 1st derivative function, called respectively der_med_ and der_max_, were then used to determine P_max_ from P_t.ave_ as follows:

$$\:{P}_{max}=\:\frac{{P}_{ave}\:\bullet\:\:{der}_{max}}{{der}_{med}}$$

Daily carotenoid productivity (P_car_) was calculated with the following equation:

$$\:{P}_{car}=\:\frac{{C}_{t1}-\:{C}_{t0}}{t1-t0}$$

Where t1 and t0 are two specific time points (days) and C_t1_, C_t0_ are the volumetric carotenoid concentration (mg L^− 1^) respectively at t1 and t0.

### Cell concentration determination

To determine cell concentration, the cell suspension was collected and diluted to approximately 1 × 10^6^ cell/mL by ASW, around 1 × 10^5^ cell/mL by isotonic solution (ISOTON, Beckman Coulter). The cell concentration was then analyzed using a Coulter counter (Multisizer 4e, Beckman Coulter) equipped with 100 μm aperture tube.

### Pigment identification and quantification

To quantify chlorophyll a and total carotenoids (car), 5 × 10^7^ – 3 × 10^8^ cells were centrifuged, and pigments were extracted by overnight incubation with dimethyl sulfoxide (DMSO) at room temperature. Next,95% acetone (buffered with sodium carbonate) was added to the DMSO extract to obtain an 80% acetone final solution. Extract samples were centrifuged at max speed for 10 min at 4 °C. Their absorption spectra were measured with Jasco UV3000 spectrophotometer: chlorophyll and carotenoids concentration was then determined by absorption spectra reconstructed as previously described [37]. HPLC then analysed pigment extracts: Reverse-phase HPLC was conducted as described in [38] and [30]. In particular, an HPLC system equipped with a C18 column was used, employing a 15-min gradient of ethyl acetate (0–100%) in acetonitrile–water–triethylamine (9: 1: 0.01, vol/vol/vol) at a flow rate of 1.5 mL/min. Pigment detection was conducted with a Thermo Fisher 350–750 nm diode array detector. Ketocarotenoid peaks were identified by comparing their retention times and spectra with those of commercially available standards (CaroteNature GmbH, Munsingen, Switzerland).

### Photosynthetic parameters

Effective PSII quantum yield (YII), Electron Transport Rate (ETR), and Non-Photochemical Quenching (NPQ) were measured as described in [39] and calculated as described by [40, 41]. In vivo chlorophyll fluorescence was measured with a Dual PAM-100 fluorometer (Walz, Effeltrich, Germany) at room temperature (RT) using a saturating light at 4500 µmol m^− 2^ s^− 1^ and actinic light of 150–300 – 600–1200 and 2400 µmol m^− 2^ s^− 1^.

### Statistical analysis

All data processing and statistical analysis were done using Excel and Origin 2018 software. No statistical predetermination of sample size was applied. Sample sizes were chosen based on experience. Statistical analysis was performed by using a two-sided Student’s t-test or ANOVA with post-hoc Tukey test in the case of multiple comparisons considering in both cases significant differences with p-values (p) lower than 0.05.

## Results

### Expression of chlamydomonas reinhardtii β-ketolase in *Nannochloropsis oceanica*

Canthaxanthin biosynthesis requires ketolation of beta-carotene (Fig. 1a). To obtain a strain of *N. oceanica* with increased canthaxanthin content, we designed a construct for the heterologous expression of a β-carotene ketolase from *Chlamydomonas reinhardtii* (CrBKT). The truncated version of CrBKT, with 166 residues removed from the C-terminus, has been previously used for efficient expression in *E. coli* [42] and in the green algae *C. reinhardtii* [30] and was therefore selected for heterologous expression of BKT in *N. oceanica*. The *CrBKT* gene sequence was optimized for *N. oceanica* codon usage, and an endogenous chloroplast target peptide was added in the N-terminal (violaxanthin/chlorophyll a-binding protein VCP1) [16]. Preliminary CrBKT expression was attempted in a wild-type (WT) strain by random insertion in the genome of the optimized *CrBKT* gene under the control of VCP promoter using zeocin resistance as selection marker [43]. However, no clear phenotypes were observed in the transformed lines. To overcome this limitation, a simpler screening procedure was adopted changing the background strain and using homologous recombination. In previous work, a landing pad (*LP*) strain was created with a fluorescence protein (tdTomato) inserted at a highly transcribed site: by homologous recombination, it is thus possible to substitute the tdTomato to (i) ensure high gene expression, using the transcriptional activity of RNA polymerase I (P_PolI_) in combination with an internal ribosome entry site (IRES) for translation and (ii) facilitate the screening pipeline using negative fluorescence screening to pick those lines where tdTomato was substituted with the gene of interest [17]. The construct adopted is described in detail in the Methods section. *LP* showed the same growth rate and pigment composition as the wild-type strain, indicating that tdTomato insertion did not significantly affect growth kinetic, biomass or pigment productivity (Additional file 1: Figure S1). *LP* strain thus was transformed with *BKT* construct, inducing homologous recombination at the tdTomato insertional locus (Fig. 1b).

**Fig. 2:**
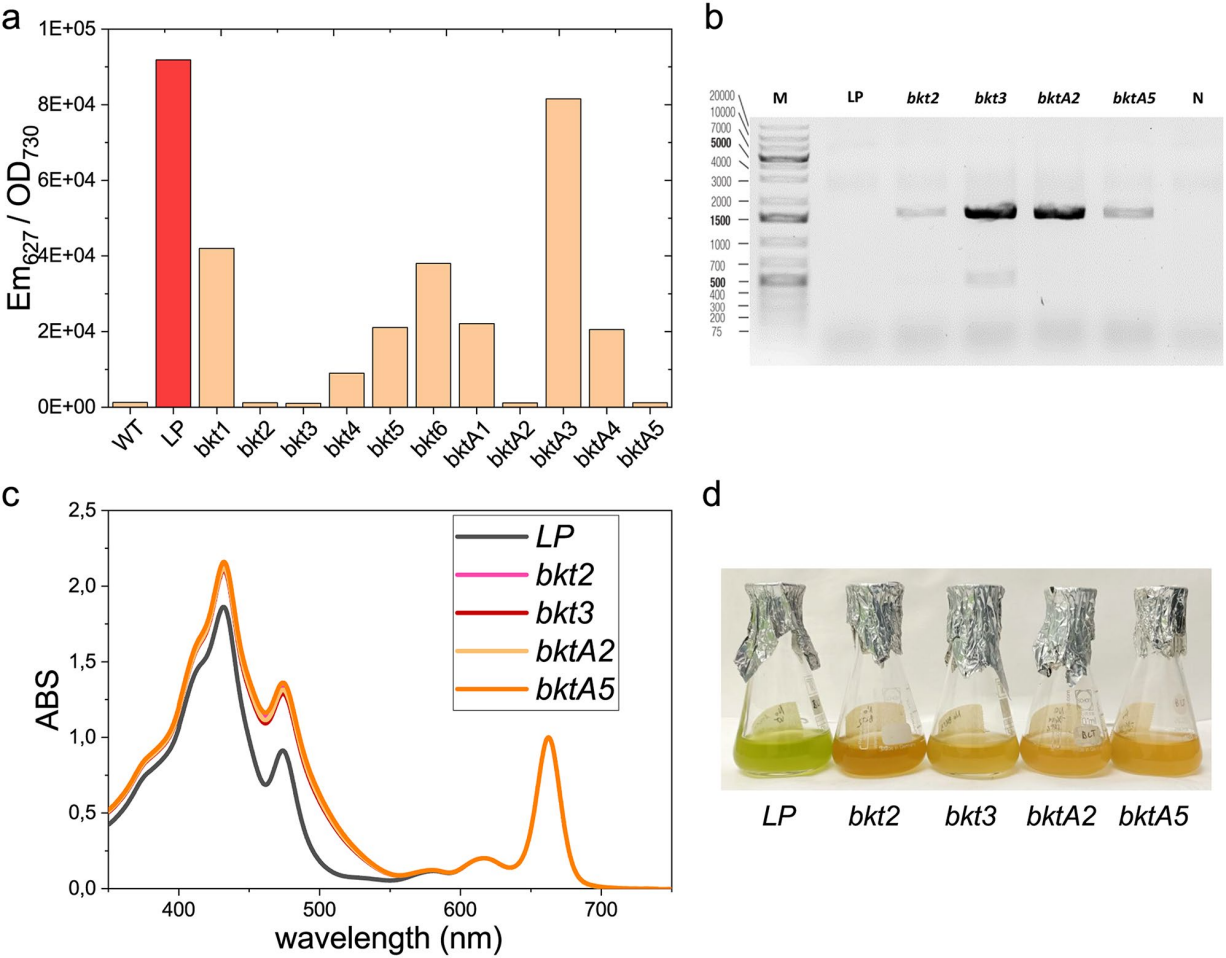
Screening of *Nannochloropsis oceanica* lines expressing CrBKT. (**a**) Fluorescence screening for the presence of tdTomato to select positive transformant candidates. (**b**) PCR on bkt-transformants to confirm correct insertion of the BKT construct in *LP* strain. (**c**) Spectra of acetone extracts from *LP* background and *bkt-* transformants. (**d**) *LP* and *bkt* transformants grown at 40 µmol m^− 2^s^− 1^ in ASW medium

Transformed colonies were first selected on antibiotic resistance on selective plates. The resistant colonies were subsequently screened for the loss of tdTomato fluorescence emission due to the homologous recombination at the target site. Four lines (*bkt2*, *bkt3*, *bktA2*, *and bktA5)* resulted in tdTomato fluorescence emission far lower than *LP* and comparable to WT (Fig. 2a), suggesting correct insertion of the *BKT* construct. PCR analysis confirmed the presence of the *CrBKT* gene in these lines (Fig. 2b). *LP* and the selected transformant lines were cultured in liquid medium in flasks at 40 µmol m^− 2^ s^− 1^ to analyze their pigment content. Pigment extracts from the transformed lines were characterized by a shoulder above 500 nm, which was absent in the pigment extract from the background strain *LP* (Fig. 2c). Indeed, canthaxanthin is characterized by a red-shifted peak compared to other carotenoids found usually in *N. oceanica*, such as violaxanthin, vaucheriaxanthin, zeaxanthin, or beta-carotene [44]. Thus, the shoulder observed in the mutated lines may indicate ketocarotenoid accumulation. The change in carotenoid composition was also evident from the orange-red color of the transformant lines compared to the *LP* background strain (Fig. 2d).

**Fig. 3:**
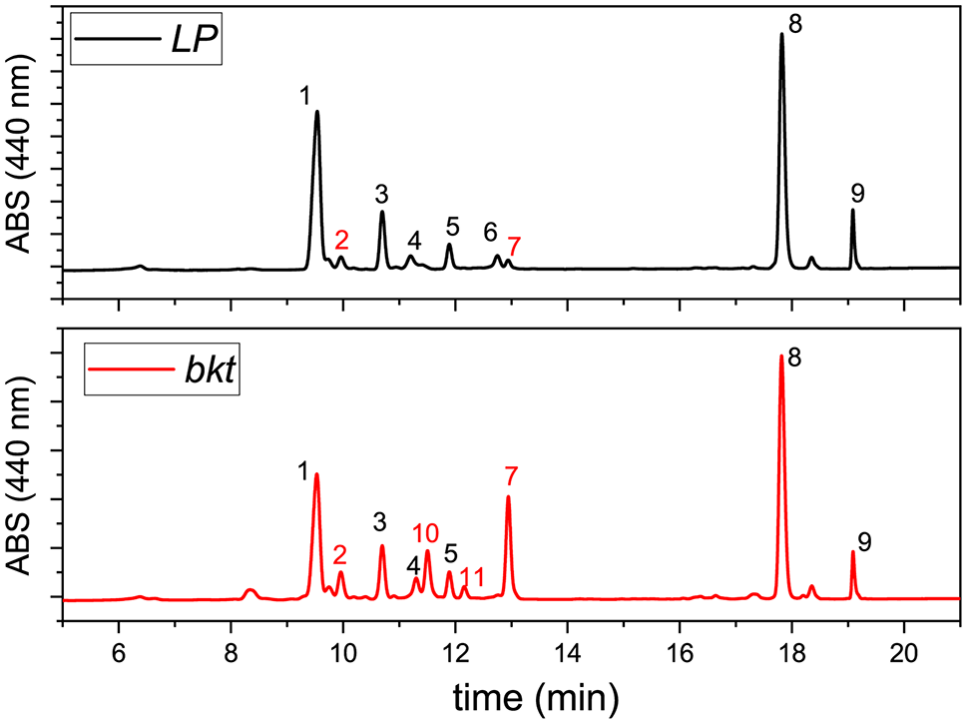
HPLC chromatogram of pigment extracts of *LP* and *bkt strains.* 1: violaxanthin, 2: astaxanthin, 3 vaucheriaxanthin, 4 antheraxanthin, 5 vaucheriaxanthin ester, 6 zeaxanthin, 7 canthaxanthin, 8 chlorophyll a, 9 β-carotene, 10 adonirubin, 11 adonixanthin. The different peaks were identified by comparing retention times and spectra to commercially available standards (CaroteNature GmbH, Munsingen, Switzerland)

Because the absorption spectra of the pigment extracts and the chlorophyll and carotenoid content per cell were almost identical across the different selected lines (Additional file 1: Figure S2), *bkt2* was chosen for subsequent analysis. Pigment extracts were then analyzed by HPLC (Fig. 3; Table 1).

**Table 1:** Pigment analysis of *bkt* lines compared to *LP* background

				carotenoids/total carotenoids									
	chl (fmol)/cell	car(fmol)/cell	chl/car	viola	vau	antera	zea	β car	asta	adonir	adonix	cantha	keto
**LP**	0.095 ± 0.002	0.050 ± 0.001	1.91 ± 0.05	0.57 ± 0.03	0.18 ± 0.02	0.06 ± 0.01	0.05 ± 0.01	0.07 ± 0.02	0.05 ± 0.01	-	-	0.03 ± 0.01	0.07 ± 0.02
***bkt***	0.095 ± 0.005	0.075 ± 0.004*	1.26 ± 0.03*	0.28 ± 0.04*	0.12 ± 0.01*	0.05 ± 0.01	-	0.05 ± 0.01*	0.08 ± 0.01*	0.14 ± 0.01*	0.01 ± 0.02*	0.26 ± 0.04*	0.49 ± 0.01

Pigment content was determined in cells grown at 40 µmol m^− 2^s^− 1^ in ASW medium for 4 days. Chlorophyll and carotenoids per cell are expressed as femtomole per cell (fmol/cell). Values marked with * indicate significant differences from *LP* (*P* < 0.05). Chl: chlorophyll; Car: carotenoids; viola: violaxanthin; vau: vaucheriaxanthin; antera: anteraxanthin; zea: zeaxanthin; β-car: β-carotene; asta: astaxanthin; adonir: adonirubin; adonix: adonixanthin; cantha: canthaxanthin; keto: total ketocarotenoids considering the values obtained for astaxanthin, adonirubin, adonixanthin and canthaxanthin. Data are expressed as means ± standard deviation (*n* ≥ 2). Raw data are reported in the additional file Table S1 and Table S2

Both *LP* and *bkt* had the same chlorophyll content per cell, while the carotenoid content in the *bkt* transformant lines increased by around 50%. These changes resulted in a lower chlorophyll/carotenoids ratio in *bkt* lines (1.26 ± 0.03 compared to 1.91 ± 0.05 in the *LP* strain). This change was also reflected in the higher absorption in the 350–550 nm range, normalized to the chlorophyll absorption peak at around 670 nm (Fig. 2c). The main carotenoids present in the *LP* were violaxanthin (more than 50% of the total) and vaucheriaxanthin, along with smaller amounts of antheraxanthin, zeaxanthin, and β carotene (Table 1). *LP* also accumulated minor quantities of ketocarotenoids like astaxanthin and canthaxanthin, accounting for roughly 7% of the total carotenoids. In contrast, the *bkt* line showed a significant shift in carotenoid distribution with ketocarotenoids comprising 50% of the total pigments. The main ketocarotenoid was canthaxanthin, which reached 26% of the total carotenoids, followed by adonirubin, astaxanthin, and traces of adonixanthin.

**Fig. 4:**
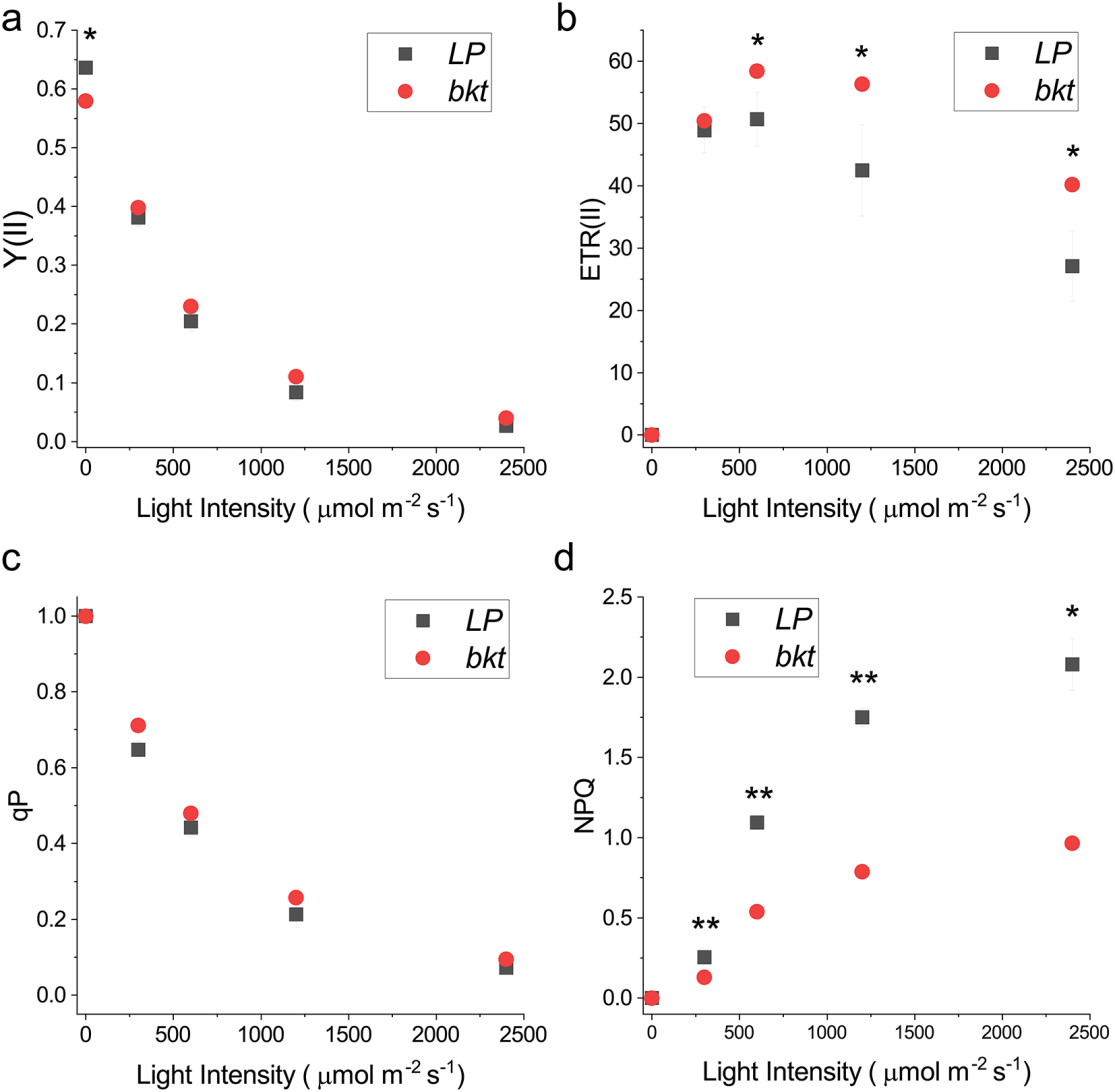
Photosynthetic parameters measured in *LP* and *bkt* lines. (**a**) Photosystem II Photochemical quantum yield (Y(II)), (**b**) Electron Transport Rate (ETR), (**c**) Non-photochemical quenching (NPQ) and (**d**) photochemical quenching (qP) were measured at different actinic lights on dark adapted cells. Data are expressed as means ± standard deviation (*n* = 3). Significantly different values are indicated with *

The consequence of changes in pigment content and accumulation on the photosynthetic activity of the *bkt* line was assessed by measuring the photosynthetic parameters Fv/Fm, Y(II), ETR, and NPQ (Fig. 4).

Fv/Fm represents the maximum photochemical efficiency of Photosystem II: a reduced Fv/Fm was observed in the *bkt* line (Fig. 4a at zero light intensities), suggesting a partial negative effect of ketocarotenoids accumulation on PSII activity. Y(II) represents the PSII photochemical activity under different light intensities: in this case, a statistically significant increase in Y(II) in the *bkt* line was measured at higher irradiances (Fig. 4a). Similarly, qP (photochemical quenching), representing the fraction of light energy used for photochemical reactions, was slightly increased in the *bkt* line compared to the background (Fig. 4b). Moreover, the ETR of PSII was increased in the *bkt line* at light intensities above 500 µmol m^− 2^s^− 1^ (Fig. 4c), suggesting an increased tolerance to high light intensity. Nevertheless, the most evident difference between *LP* and *bkt* line was observed for NPQ (Non-photochemical quenching). The *bkt* line exhibited strongly reduced NPQ at different light intensities. Even at high irradiances, such as 5400 µmol m^− 2^ s^− 1^, the NPQ in the bkt line was lower than that in the *LP* case (Fig. 4d). This result is consistent with previous findings in *C. reinhardtii* strains engineered to express BKT enzyme, resulting in being essentially impaired in NPQ induction [44].

### Biomass and ketocarotenoids yield in different growth conditions

**Fig. 5:**
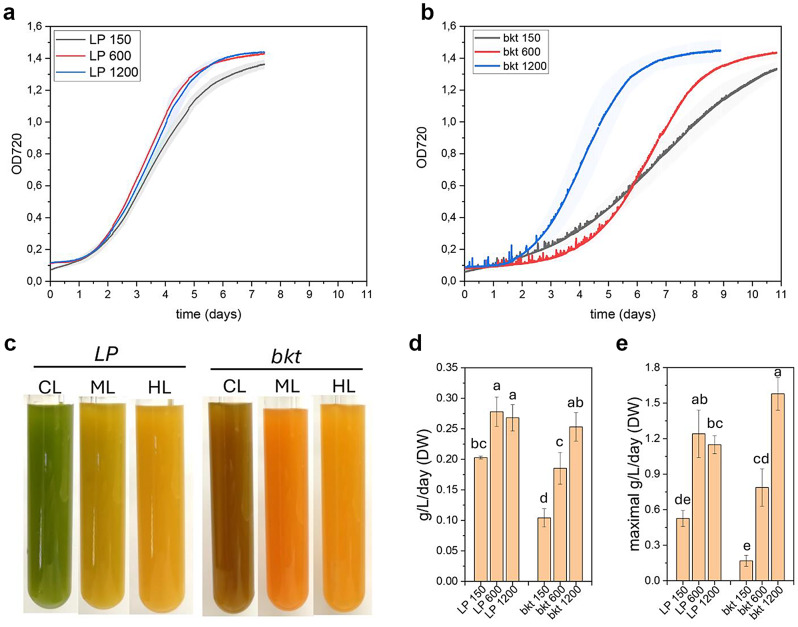
Biomass accumulation in *LP* and *bkt* lines at different light intensities. Growth curve of *LP* (**a**) and *bkt* (**b**) strain in airlifted photobioreactors at 150, 600 and 1200 µmol m^− 2^s^− 1^. Growth curves were obtained monitoring OD at 720 nm (**c**) Representative pictures at the end of the growth. Biomass productivities (**d**) and maximal productivity (**e**) obtained from *LP* and *bkt* grown as reported in panels **a** and **b**. Data are expressed as means ± standard deviation (*n* = 3). Significantly different values are indicated with different letters

To compare the growth kinetics and calculate the ketocarotenoids yield of *LP* and *bkt* line, both were cultivated in an air-lifted bioreactor under three different light intensities, 150, 600, and 1200 µmol m^− 2^s^− 1^ at 24° C providing air-enriched with 3% CO_2_ (Fig. 5). Growth curves were continuously monitored by measuring optical density at 720 nm. For the background strain *LP*, cells grown at 150 µmol m^− 2^s^− 1^ showed a lower growth rate compared to higher light intensities, while at 600 and 1200 µmol m^− 2^s^− 1,^ the growth kinetics were similar. All the *LP* samples reached the stationary phase after six days of cultivation. In contrast, the growth kinetics for the *bkt* line varied based on the irradiance to which the cells were exposed. At 150 µmol m^− 2^s^− 1,^ the *bkt* strain showed a slower growth rate than its background strain, reaching the stationary phase only after ten days. At 600 µmol m^− 2^s^− 1^, the *bkt* strain showed an extended initial lag phase but then entered an exponential phase with a growth rate comparable to *LP*. At 1200 µmol m^− 2^s^− 1^, the lag phase was like the *LP* case, but the *bkt* line was characterized by a faster growth rate during the exponential phase. At the end of the growth, the dry weight of the biomass produced was measured to calculate the average biomass productivity (Fig. 5). The *LP* strain showed an average productivity of 0.20 g L^− 1^d^− 1^ at 150 µmol m^− 2^ s^− 1^ that increased to 0.28 g L^− 1^ d^− 1^ at 600 µmol m^− 2^ s^− 1^ and remained similar at 1200 µmol m^− 2^ s^− 1^ (0.27 g L^− 1^d^− 1^). In the case of *the bkt line*, average biomass productivity was lower than in *LP* at control or medium light intensities, respectively ~ 50% and ~ 65% at 150 µmol m^− 2^ s^− 1^ and 600 µmol m^− 2^ s^− 1^ compared to their background. However, at 1200 µmol m^− 2^ s^− 1^, the biomass accumulated by the *bkt* transformant was similar to that of the *LP* strain but with higher maximum biomass productivity of ~ 1.6 g L^− 1^d^− 1^ compared to ~ 1.2 g L^− 1^d^− 1^ for the *LP* strain.

Pigments amount and distribution were calculated at the end of the growth curves by HPLC (Table 2). Chlorophyll and carotenoid content radically changed at different light intensities, showing a reduction on a per-cell basis in both *LP* and *bkt* lines at higher irradiances (Table 2).

**Table 2:** Pigment content at the different light intensities

			carotenoids/total carotenoids									
	Chl /cell	Car /cell	viola	vau	antera	zea	β car	asta	adonir	adonix	cantha	keto
LP 150	0.069 ± 0.016^a.c^	0.040 ± 0.007^a^	0.53 ± 0.04^a^	0.26 ± 0.01^a^	0.04 ± 0.01	0.03 ± 0^a^	0.06 ± 0^a^	0.05 ± 0.01^a.c^	0 ± 0^a^	0 ± 0^a^	0.03 ± 0.01^a^	0.07 ± 0.02^a^
LP 600	0.015 ± 0.006^b^	0.017 ± 0.004^b^	0.42 ± 0.03^a.b^	0.18 ± 0.03^a.b^	0.07 ± 0	0.06 ± 0^b^	0.06 ± 0^a^	0.15 ± 0.04^b.c^	0 ± 0^a^	0 ± 0^a^	0.06 ± 0.02^a^	0.21 ± 0.06^a.b^
LP 1200	0.010 ± 0.002^b^	0.014 ± 0.002^b^	0.39 ± 0.02^a.b^	0.15 ± 0^b^	0.06 ± 0.02	0.08 ± 0.01^c^	0.06 ± 0.01^a^	0.17 ± 0.01^b^	0 ± 0^a^	0 ± 0^a^	0.09 ± 0.01^a^	0.26 ± 0.02^b^
*bkt* 150	0.088 ± 0.026^a^	0.059 ± 0.011^c^	0.31 ± 0.01^b^	0.15 ± 0.01^b^	0.04 ± 0	0 ± 0^d^	0.04 ± 0^b^	0.09 ± 0.01^a.c^	0.06 ± 0.01^b.c^	0.06 ± 0.02^b^	0.26 ± 0.04^b^	0.46 ± 0.02^c^
*bkt* 600	0.023 ± 0.006^b.c^	0.026 ± 0.003^a.b^	0.31 ± 0.07^b^	0.15 ± 0.02^b^	0.04 ± 0	0 ± 0^d^	0.02 ± 0^c^	0.08 ± 0.01^c^	0.07 ± 0.01^b^	0.06 ± 0.02^b^	0.27 ± 0.04^b^	0.48 ± 0.08^c^
*bkt* 1200	0.007 ± 0.005^b^	0.014 ± 0.003^b^	0.32 ± 0.06^a.b^	0.17 ± 0.04^b^	0.04 ± 0.03	0 ± 0^d^	0.01 ± 0^c^	0.13 ± 0.02^b.c^	0.04 ± 0^c^	0.03 ± 0.02^a.b^	0.20 ± 0.02^b^	0.45 ± 0.01^c^

Pigment content was determined in cells grown in air-lifted photobioreactors at different light intensities (150, 600, 1200 µmol m^− 2^s^− 1^) in ASW medium for 7–11 days. Chlorophyll and carotenoids per cell are expressed as femtomole (fmol). X Chl: chlorophyll; Car: carotenoids; viola: violaxanthin; vau: vaucheriaxanthin; antera: anteraxanthin; zea: zeaxanthin; β-car: β-carotene; asta: astaxanthin; adonir: adonirubin; adonix: adonixanthin; cantha: canthaxanthin; total ketocarotenoids considering the values obtained for astaxanthin, adonirubin, adonixanthin and canthaxanthin. Data are expressed as means ± standard deviation (*n* ≥ 2). Significant different values for *bkt* compared to *LP* are indicated with * (*p* < 0.05). Raw data are reported in the additional file table S3 and table S4

**Fig. 6:**
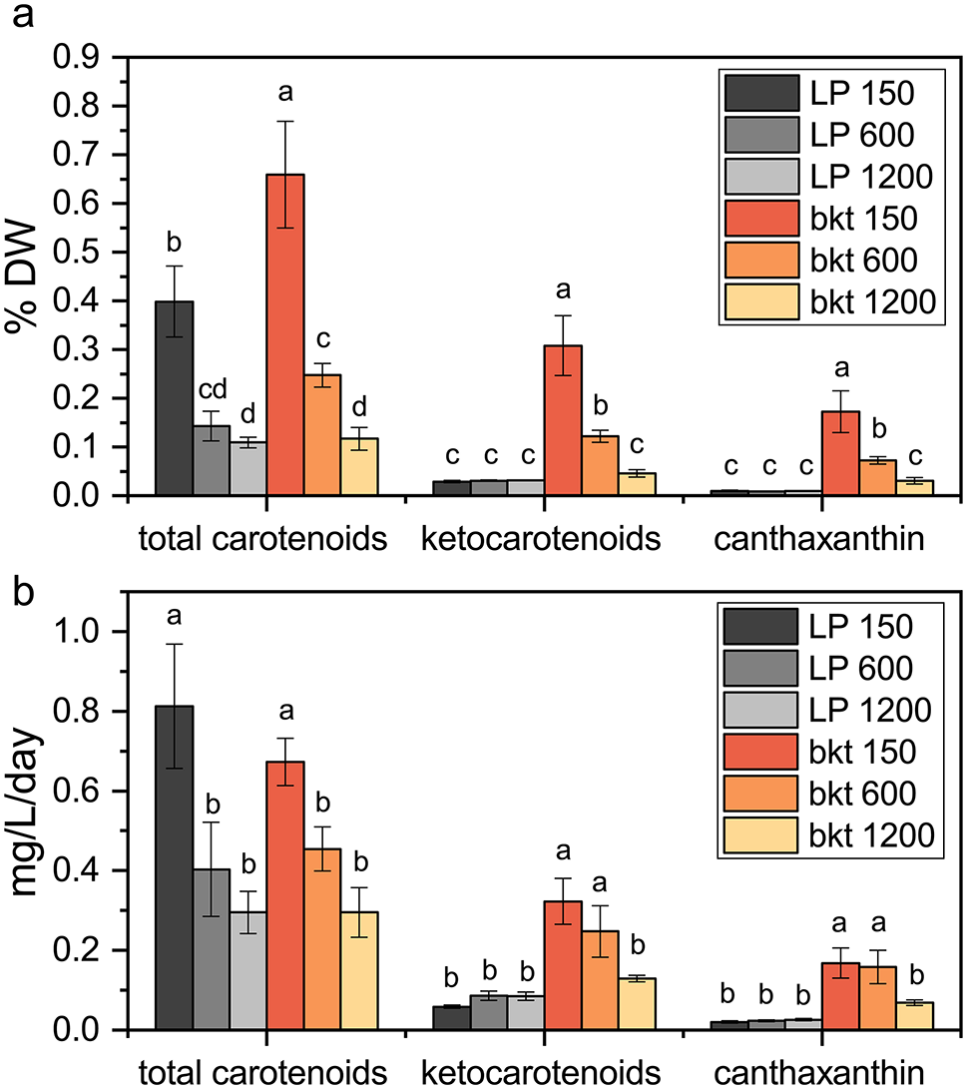
Carotenoids and ketocarotenoids productivities in *LP* and *bkt* lines. Total carotenoids, ketocarotenoids and canthaxanthin content per dry weight (**a**) and average daily productivity (**b**) at the end of the experiment described in Fig. 5. Data are expressed as means ± standard deviation (*n* = 3). Significantly different values are indicated with different letters

As previously reported for cells grown at 40 µmol m^− 2^s^− 1^ in flasks, the carotenoid content per cell was higher in the *bkt* line compared to *LP* at 150 µmol m^− 2^ s^− 1^, but not at 600 or 1200 µmol m^− 2^ s^− 1^, where similar carotenoids per cell ratios were measured. Similar results about carotenoid content were also evident on a dry weight basis (Fig. 6a): both *LP* and *bkt* line were characterized by reduced carotenoid content per dry weight as light intensity increased. While at 150 and 600 µmol m^− 2^ s^− 1^, the *bkt* line showed higher carotenoid content than the background strain. At 1200 µmol m^− 2^s^− 1^, the fraction of carotenoid per dry weight were similar. At all three light intensities, the *bkt* line accumulated ~ 45% of its carotenoids as ketocarotenoids, more than half of which being canthaxanthin. The *LP* strain increased its ketocarotenoids content with increasing light intensity, reaching only 26% of total carotenoids at the highest light intensity herein used (1200 µmol m^− 2^s^− 1^). In the *LP strain*, the main ketocarotenoid accumulated was astaxanthin, while canthaxanthin consistently constituted less than 10% of total carotenoids. Total ketocarotenoid yields, especially canthaxanthin, were firmly higher in the transformant line. The transformant line reached a maximum productivity of ketocarotenoids of ~ 0.32 mg L^− 1^d^− 1^ when grown at 150 µmol m^− 2^s^− 1^ while the background strain reached only ~ 0.08 mg L^− 1^d^− 1^ when grown at 600 or 1200 µmol m^− 2^s^− 1^. Similar results were obtained for canthaxanthin with maximal productivity of ~ 0.17 mg L^− 1^d^− 1^ and ~ 0.02 mg L^− 1^d^− 1^ for *bkt* and *LP*, respectively. The *bkt* transformant showed a canthaxanthin yield eight times higher than the parental line, reaching ~ 0.2% of dry weight at 150 µmol m^− 2^ s^− 1^. The other ketocarotenoids accumulated in *bkt* were astaxanthin, adonirubin, and adonixanthin reaching a maximum level of 0.07%, 0.05%, and 0.04% per dry weight, respectively, at 150 µmol m^− 2^s^− 1^. The substantial decrease in pigment content at higher light intensities led to the conclusion that the control light of 150 µmol m^− 2^s^− 1^ is the most suitable to trigger ketocarotenoids and canthaxanthin production in *N. oceanica* (Fig. 6b).

### Ketocarotenoids and canthaxanthin productivity

**Fig. 7:**
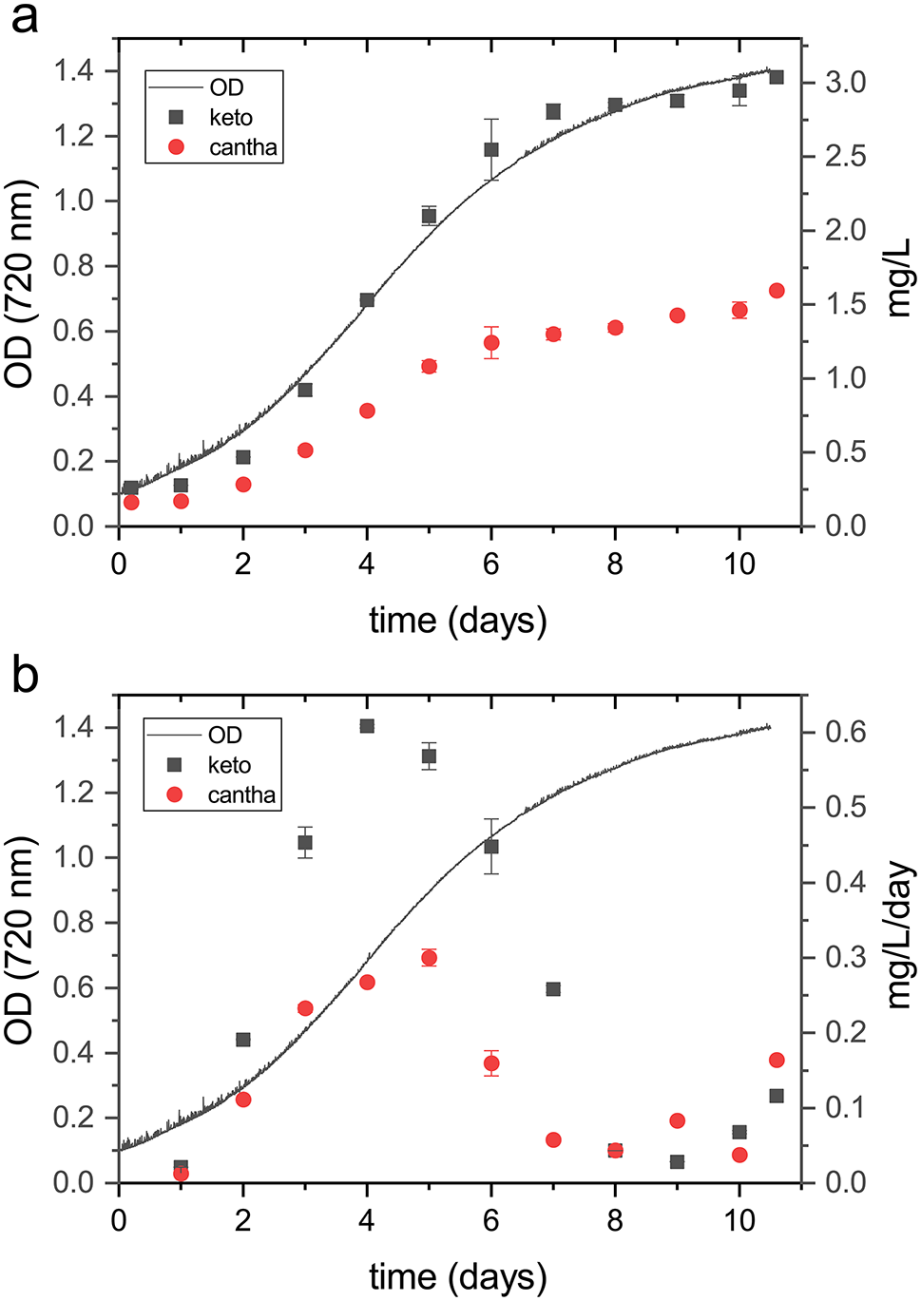
Ketocarotenoids and canthaxanthin productivity during the growth curve. Volumetric production (**a**) and volumetric productivity (**b**) correlated with optical density measurements at 720 nm during the growth 150 µmol m^− 2^s^− 1^. Data are expressed as means ± standard deviation (*n* = 3)

Ketocarotenoids and canthaxanthin accumulation were monitored upon cell cultivation at 150 µmol m^− 2^ s^− 1^ by sampling cells at different time points. The stationary growth phase was reached after ten days at an optical density of 1.4, corresponding to a cellular concentration of 5.3*10^8^ cells per ml. Maximal volumetric production was observed at the end of the experiment with an observed value of ~ 3 mg/L of ketocarotenoids and 1.6 mg/L of canthaxanthin. Volumetric ketocarotenoid and canthaxanthin productivity were correlated with the algal growth phase, and showed a reduction as growth slowed (Fig. 7). Ketocarotenoids productivity peaked on the fourth day with a calculated productivity of 0.6 mg L^− 1^ day^− 1^. Canthaxanthin productivity reaches its peak on the fifth day, with a calculated productivity of 0.3 mg L^− 1^ day^− 1^ (Fig. 7).

Different stress treatments, like nutrient deprivation and light stress, can increase carotenoid content in different microalgae species [30, 45–48]. A two-stage growth approach was tested in *the bkt line* to evaluate the optimal stress condition for increasing carotenoid yield. In the first cultivation phase, conditions were optimized for biomass accumulation, while the second phase involved applying stress conditions to potentially increase the pigment content (Additional file 1: Figure S3). At the end of growth under light conditions of 150 µmol m^− 2^ s^− 1^, the *bkt* microalgae were exposed to extremely high light (3000 µmol m^− 2^ s^− 1^) or resuspended in nitrogen free-medium to induce nitrogen starvation. After two days, HPLC was used to analyze the carotenoid content. Nitrogen deprivation did not modify the ketocarotenoids fraction of total carotenoids; however, the overall carotenoid content per cell was reduced; resulting in a lower ketocarotenoids content per dry weight under nitrogen starvation compared to control conditions (Additional file 1: Figure S3). In contrast, high light stress increased the ketocarotenoid fraction of total carotenoids by ~ 30%. However, even in this case, the light stress also decreased the total carotenoid content per cell. Consequently, the ketocarotenoids content per dry weight was again lower than the control condition. Thus, a two-stage growth approach was effective in further accumulating ketocarotenoids in *N. oceanica*, cultivation under non-stress conditions proved to be the most effective condition for ketocarotenoids and canthaxanthin accumulation.

## Discussion

*Nannochloropsis* species naturally accumulate high-value ketocarotenoids such as canthaxanthin and astaxanthin, though typically in traces unless triggered by high light stress [47]. In the background strain of *N. oceanica LP*, herein adopted, astaxanthin biosynthesis was triggered under high light. At 40 µmol m^-2^ s^-1^, astaxanthin was 5% of total carotenoids respectively; at 1200 µmol m^-2^ s^-1^, this percentage increased to 17% (Table 1). Canthaxanthin was instead accumulated only in minimal amounts, either in low or high light (up to 9% of total carotenoids).

A possible solution to improve ketocarotenoid accumulation is the heterologous expression of the BKT enzyme to increase canthaxanthin biosynthesis from β-carotene and astaxanthin production from zeaxanthin (Fig. 1a). Over-expression of the optimized CrBKT enzyme in *C. reinhardtii* converted 50% of carotenoids into astaxanthin and more than 70% into other ketocarotenoids [30]. In this work, *CrBKT* gene was inserted in *N. oceanica* in a newly developed strain that guarantees high levels of transcription and translation of genes of interest. This new *LP* strain showed great potential for the expression of heterologous genes and allowed a robust CrBKT expression. Even under low light, the *bkt* strain converted 50% of its carotenoids into ketocarotenoids. The major ketocarotenoid present in the *bkt* line was canthaxanthin, which reached 26% of total carotenoids and, under optimal conditions, ~ 0.2% of dry weight, representing a substantial increase in canthaxanthin production in *Nannochloropsis*. Unlike *C. reinhardtii*, where CrBKT expression was sufficient to trigger most of the produced ketocarotenoids into astaxanthin, the *N. oceanica bkt* lines primarily accumulate canthaxanthin. This ketocarotenoid was steadily accumulated even under low light conditions without the need for a stressing phase to trigger its biosynthesis. Recombinant expression in *E. coli* showed that the CrBKT enzyme has a high affinity for β carotene or zeaxanthin with conversion rates above 80% [42]. Thus, the reduced astaxanthin accumulation cannot be attributed to the specificity of CrBKT. In *N. oceanica*, the accumulation of zeaxanthin, especially under low light conditions, is limited, suggesting that the primary substrate for the CrBKT is β-carotene, which is converted into canthaxanthin through ketolation. The endogenous hydroxylases of *N. oceanica* may have a low affinity for ketocarotenoids, which could hinder the ability to work efficiently on canthaxanthin. As shown in Table 2, N. *oceanica* accumulates astaxanthin under high-light conditions, during which the xanthophyll cycle also synthesizes zeaxanthin. The natural substrate for *Nannochloropsis* endogenous ketolase, still unknown, may be zeaxanthin, which can be directly converted to astaxanthin without the need to go through canthaxanthin hydroxylation. These results demonstrate that the endogenous hydroxylase activity is the limiting step for astaxanthin biosynthesis. This conclusion is also supported by the accumulation of adonirubin, an intermediate between canthaxanthin and astaxanthin (Fig. 1a), which showed hydroxylation on only one of the β rings, modification that was not detected in *C. reinhardtii* transformed strains. This allowed the accumulation of canthaxanthin in a high percentage in *N. oceanica*, representing a novel biological source for this pigment with specific applications in poultry feed.

Carotenoids are essential for optimal light harvesting in photoautotrophic organisms. Any changes in their distribution may destabilize the photosystems, reducing the light use efficiency, impairing biomass accumulation. Many mutants in plants and microalgae with altered xanthophyll composition have showed reduced light harvesting efficiency [33, 49–52]. Violaxanthin is the primary carotenoid present in *N. oceanica* and its antenna system. In the *bkt* line, the percentage of violaxanthin relative to total carotenoids was significantly decreased in cells grown at 40 µmol m^-2^ s^-1^ (Table 1) or 150 µmol m^-2^ s^-1^ (Table 2): reduced violaxanthin content may influence light use efficiency at low irradiances. Indeed, the *bkt* strain had a lower growth rate at 150 µmol m^-2^ s^-1^ compared to the parental strain and accumulated less biomass. This behavior was different for the *C. reinhardtii* CrBKT expressing lines, which under low-light conditions showed a growth curve similar to the parental strain. It is worth noting that in *C. reinhardtii* a decrease of chlorophyll content per cell was observed upon overexpression of CrBKT [30]: this pale phenotype may compensate for decreased light harvesting efficiency in CrBKT expressing lines, favoring a better light penetration within the tubes, as already demonstrated in different strains with reduced pigment content [44, 53–55]. However, this was not the case for *N. oceanica* strains herein engineered, where *bkt* line and *LP* background shared similar chlorophyll contents per cell (Table 1). By the way, even in *N. oceanica*, the CrBKT expressing line improved its biomass yield under higher light intensity. At 1200 µmol m^-2^ s^-1,^ the *bkt* transformant showed a higher growth rate and maximal productivity than the *LP* background. Ketocarotenoids are potent antioxidant molecules that scavenge reactive oxygen species, and act as a protective barrier against the oxidation of lipids, pigments, and photosynthetic complexes. Higher amounts of canthaxanthin and other ketocarotenoids can improve tolerance to ROS generated under high light. Accordingly, the photosynthetic properties of the *bkt* line showed an increased ETR and reduced NPQ at higher light intensities compared to the *LP* line, supporting increased high light tolerance upon BKT expression and ketocarotenoids accumulation. A similar effect was seen in *C. reinhardtii* or *Synechococcus* transformant expressing BKT enzymes characterized by improved high light tolerance, reduced photoinhibition, and improved biomass productivity at high irradiances [23, 44]. According to these results, engineered ketocarotenoids accumulation could be a promising strategy to enhance *N. oceanica* growth at stronger irradiances.

*N. oceanica* undergoes substantial changes in pigment content when adapted to different light intensities: at 1200 µmol m^-2^ s^-1,^ chlorophyll content dropped to one-tenth, and carotenoid content decreased 5-fold (Table 2). When exposed to higher light intensity, microalgae decrease pigment content to avoid excess light absorption and photoinhibition [56–58]. Because of these changes, a dichotomy in canthaxanthin productivity was balanced between these two opposite effects: increased growth rate but lower ketocarotenoids per cell at higher light intensities (Fig. 6). This is a relevant aspect from a productivity point of view because it means that the highest canthaxanthin productivity could be reached in low-light conditions. The illumination energy is one of the major operative costs in a photobioreactor because substantial light intensity is needed to obtain adequate productivity. In this case, the energy demand would be lower. Besides, microalgae cultivation plants must be designed considering light penetration in dense cultures; many implants have a depth of a few centimeters to avoid the shading of the inner layers. The lower light demands of this strain would allow the design of bioreactor with higher depths to improve per-area productivity compared to the cultivation required for other algae.

Previous work reported canthaxanthin accumulation in *Haematococcus* strains below 0.05% of dry weight [59–62]: canthaxanthin is indeed usually accumulated in *Haematococcus* in traces being a precursor for astaxanthin, the main carotenoid found in stress conditions. In our cases, it was possible to reach up to ~ 0.2% of canthaxanthin per dry weight without the need for any stress phase to trigger carotenogenesis. Lipids and carotenoids can be increased by applying nutritional stress (nitrogen deprivation) during the stationary phase, and high light stress induces carotenoid accumulation. These strategies did not work to trigger ketocarotenoids productivity in the *N. oceanica bkt* strains. Neither nitrogen deprivation nor high light stress caused increased volumetric productivity of ketocarotenoids or canthaxanthin (Fig. 7), suggesting that single-phase cultivation is the best option to produce ketocarotenoids by the *bkt* lines herein generated. A continuous/semicontinuous system could be preferred to increase the productivity in a bioreactor, maintaining the microalgal cells in the exponential phase at their maximum productivity peak. Moreover, a possible fed-batch strategy could be applied to provide sufficient nutrients as nitrates and phosphates, to boost biomass and canthaxanthin productivity. Additionally, directing the metabolic flux toward carotenoid biosynthesis by overexpressing enzymes in this pathway could be an additional strategy to increase the canthaxanthin content in *bkt* cells. Further improvement in overall carotenoid accumulation may be obtained by overexpression of enzymes involved in the MEP pathway to boost the accumulation of the an isopentyl phosphate pool: the carbon flux through the MEP-pathway is indeed reported to be limited by DXS and GGPPS as key enzymatic bottlenecks, which can be deregulated by heterologous overexpression [63]. Similarly, carotenoid biosynthesis could be boosted by overexpression of geranylgeranyl diphosphate synthase (GGPPS), providing substrate for phytoene synthase, the key enzyme to initiate carotenogenesis and/or by overexpression of the enzymes involved in the initial step of carotenoid biosynthesis as PSY itself or lycopene cyclase. In the case of CrBKT expression in *C. reinhardtii*, the highest ketocarotenoid production was observed upon overexpression of phytoene synthase and lycopene cyclase [63]. Mutation of the beta-carotene hydroxylase enzyme could also be attempted to block zeaxanthin (and violaxanthin) biosynthesis and, consequently astaxanthin biosynthesis in the *bkt* lines. Zeaxanthin epoxidase could also be considered as a potential target for genetic modification to decrease its catalytic activity and reduce the accumulation of violaxanthin, and thus redirect the metabolic flux toward ketocarotenoids (Fig. 1). However, such mutations could have potential negative consequences on growth rate and biomass productivity, as zeaxanthin and violaxanthin are the main xanthophylls accumulated in *N. oceanica* [32, 33]. It is worth noting that during the revision process of this paper, two independent articles were published with a similar aim to boost ketocarotenoids production in *N. oceanica* by overexpression of CrBKT [64, 65]: the results reported are in line with the findings herein reported, with an improved canthaxanthin production, reaching up 0.47% thanks to further overexpression of GGPPS gene or suppressing zeaxanthin epoxidase 1 gene [64].

## Conclusions

Microalgae, thanks to their high protein and lipid content, nutritional values, and phototrophic growth, are potential replacements for food and feed products from traditional agriculture. Their efficient carbon capture and ability to thrive on non-arable land give them a pivotal role in addressing the urgent need for sustainable solutions to meet rising food demands and facilitate the transition to lower carbon emissions. For marine microalgae, like *Nannochloropsis*, there is also no competition for freshwater sources. Microalgae naturally produce many compounds of interest, including lipids, antioxidants, biopolymers, pigments, polysaccharides, and peptides. By heterologous expression of the CrBKT enzyme, it was possible to redirect the metabolic flow to ketocarotenoid production. The limited activity of endogenous beta-carotene hydroxylase limited the accumulation of astaxanthin, while high canthaxanthin production reached 0.2% of dry weight. This metabolic engineering approach offers potential applications such as formulating fish or poultry feed with high canthaxanthin content to achieve desired pigmentation, exploiting as well, its high EPA content, an omega-3 fatty acid that is another important additive in the feed industry.

## Electronic supplementary material

Below is the link to the electronic supplementary material.

Additional file 1: Sequences of CrBKT gene and oligonucleotides used in study and Figure S1-S2

## Data Availability

All data generated or analyzed during this study are included in this published article and its supplementary information files.
