# Supplementary material for: Genetic engineering of Nannochloropsis oceanica to produce canthaxanthin and ketocarotenoids
Source: Microb Cell Fact. 2024 Nov 29;23:322. doi: 10.1186/s12934-024-02599-4 (PMC11606307; doi:10.1186/s12934-024-02599-4)
Supplement: Supplementary file 1 — Additional file 1: Sequences of CrBKT gene and oligonucleotides used in study and Figure S1-S2 [file 12934_2024_2599_MOESM1_ESM.pdf]

Sequence of *CrBKT* gene optimized for *Nannochloropsis ocenica* expression.

ATGGGCCCCGGCATCCAGCCCACCTCCGCCCGCCCCTGCTCCCGCACCAAGCACTCCCGCT
TCGCCCTCCTCGCCGCCGCCCTCACCGCCCGCCGCGTCAAGCAGTTCACCAAGCAGTTCCG
CTCCCGCCGCATGGCCGAGGACATCCTCAAGCTCTGGCAGCGCCAGTACCACCTCCCCCGC
GAGGACTCCGACAAGCGCACCCTCCGCGAGCGCGTCCACCTCTACCGCCCCCCCCGCTCCG
ACCTCGGCGGCATCGCCGTCGCCGTCACCGTCATCGCCCTCTGGGCCACCCTCTTCGTCTAC
GGCCTCTGGTTCGTCAAGCTCCCCTGGGCCCTCAAGGTCGGCGAGACCGCCACCTCCTGGG
CCACCATCGCCGCCGTCTTCTTCTCCCTCGAATTTCTCTACACCGGCCTCTTCATCACCACCC
ACGACGCCATGCACGGCACCATCGCCCTCCGCAACCGCCGCCTCAACGACTTCCTCGGCCA
GCTCGCCATCTCCCTCTACGCCTGGTTCGACTACTCCGTCCTCCACCGCAAGCACTGGGAGC
ACCACAACCACACCGGCGAGCCCCGCGTCGACCCCGACTTCCACCGCGGCAACCCCAACCT
CGCCGTCTGGTTCGCCCAGTTCATGGTCTCCTACATGACCCTCTCCCAGTTCCTCAAGATCGC
CGTCTGGTCCAACCTCCTCCTCCTCGCCGGCGCCCCCCTCGCCAACCAGCTCCTCTTCATGA
CCGCCGCCCCCATCCTCTCCGCCTTCCGCCTCTTCTACTACGGCACCTACGTCCCCCACCAC
CCCGAGAAGGGCCACACCGGCGCCATGCCCTGGCAGGTCTCCCGCACCTCCTCCGCCTCCC
GCCTCCAGTCCTTCCTCACCTGCTACCACTTCGACCTCCACTGGGAGCACCACCGCTGGCCC
TACGCCCCCTGGTGGGAGCTCCCCAAGTGCCGCCAGATCGCCCGCGGCGCCGCCCTCGCC
GGCTCCGGC

Oligonucleotides used for this study.

ID	Sequence	Application
CrBKTtot_fwd	TTCTCCCGCACCCGCGGTGTTG CGATGGGCCCCGGCATCCAGC	Amplification of BKT from BKT genestrand for construction of pCcTE-BKT
CrBKTtot_rev	TCAGTGATGGTGATGGTGATGG CCGCCGGAGCCGGCGAGGGC	Amplification of BKT from BKT genestrand for construction of pCcTE-BKT
pCpTE-PolI_fwd	GGCCATCACCATCACCATCACT GATG	Amplification of pCpTE backbone from pCpTE-PolI for construction of pCcTE-BKT
pCpTE-PolI_rev	CGCAACACCGCGGGTGCG	Amplification of pCpTE backbone from pCpTE-PolI for construction of pCcTE-BKT
oCST033_fwd	AGGTAAGGAGTAGGGAGGGG	Amplification of BKT construct from pCpTE-BKT for transformation
oCST033_rev	ATCCGCCGTCACACTAATCA	Amplification of BKT construct from pCpTE-BKT for transformation
NocIRESControl_fwd	AATGTGATTTCTGCCCAGTG	Amplification of partial BKT construct from pCpTE-BKT for colony PCR and sequencing
Alpha-tub-Control_rv	TATGACGGGCACACACACAC	Amplification of partial BKT construct from pCpTE-BKT for colony PCR and sequencing
3' Blast frw	ACGTGTGGGAGAGCGCTTGA	Amplification of partial BKT construct from *bkt* transformants gDNA to confirm correct insertion
3' chr. 3 rv	TCTGCGGATGCTGCTACTTTCA	Amplification of partial BKT construct from *bkt* transformants gDNA to confirm correct insertion

Figure S1

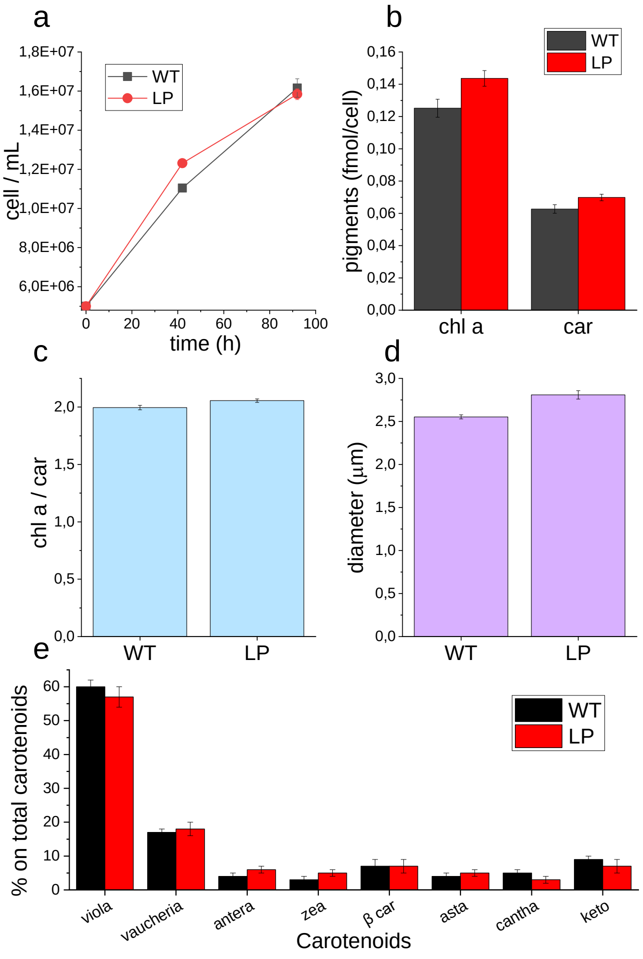

Figure S1. Growth and pigment composition of wild-type and LP strains. a) cell density at different points upon cultivation at 80 umol m^{-2} s^{-1} of wild-type (WT) and LP strain. b) cell diameter of WT and LP cells cultivated as in A. c) Chlorophyll a (chl a) and carotenoids (car) content per cells of WT and LP strain grown as reported in A. d) Chlorophyll to carotenoids ratio (chl a/car) calculated from the data reported in B. e) Distribution of the different carotenoid species in WT and LP strains as percentage on total carotenoid content. Viola: violaxanthin; vaucheria: vaucheriaxanthin; antera: anteraxanthin; zea: zeaxanthin; β-car: β-carotene; asta: astaxanthin; adonir: adonirubin; adonix: adonixanthin; cantha: canthaxanthin; total ketocarotenoids considering the values obtained for astaxanthin, adonirubin, adonixanthin and canthaxanthin. Data are expressed as means ± standard deviation (n=3). The values reported are not significantly different as evaluated by using a two-sided Student's t-test.

Figure S2

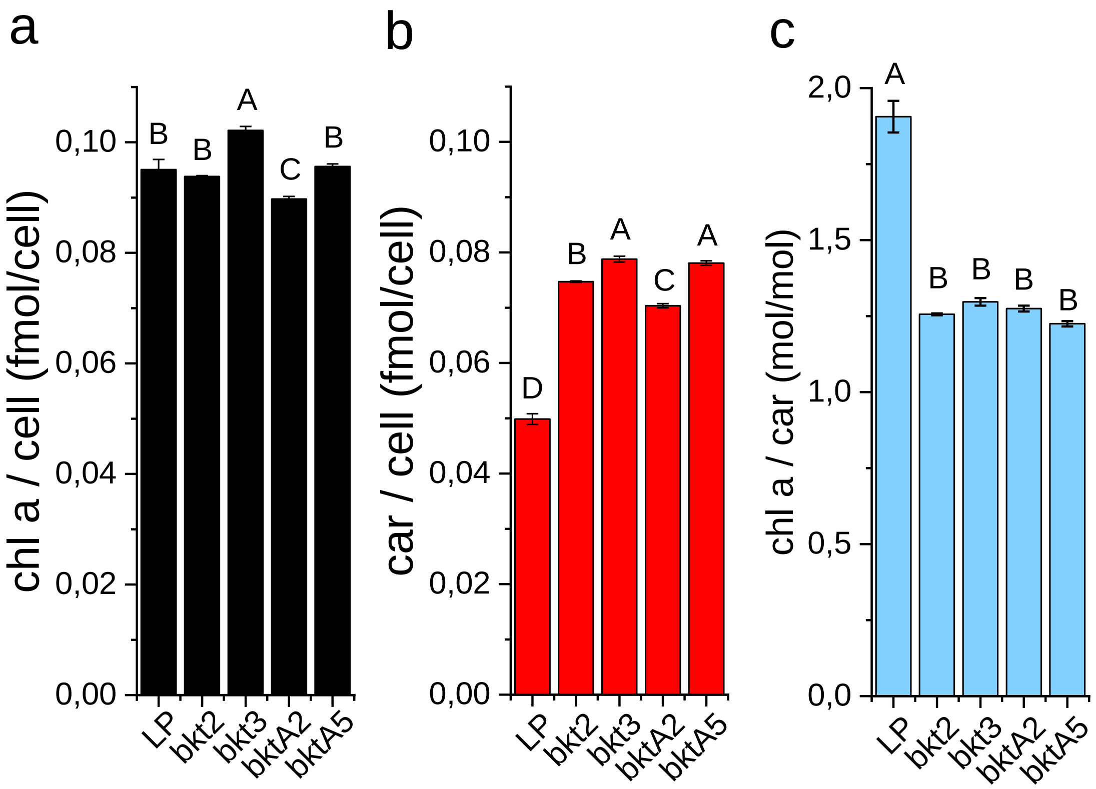

Figure S2. Pigment composition of LP and *bkt* strains. a) Chlorophyll a (chl a) and b) carotenoids (car) content per cells of LP and *bkt* strains. c) Chlorophyll to carotenoids ratio (chl a/car) calculated from the data reported in a) and b). Data are expressed as means ± standard deviation (n=2). Significantly different values are indicated with different letters.

Figure S3

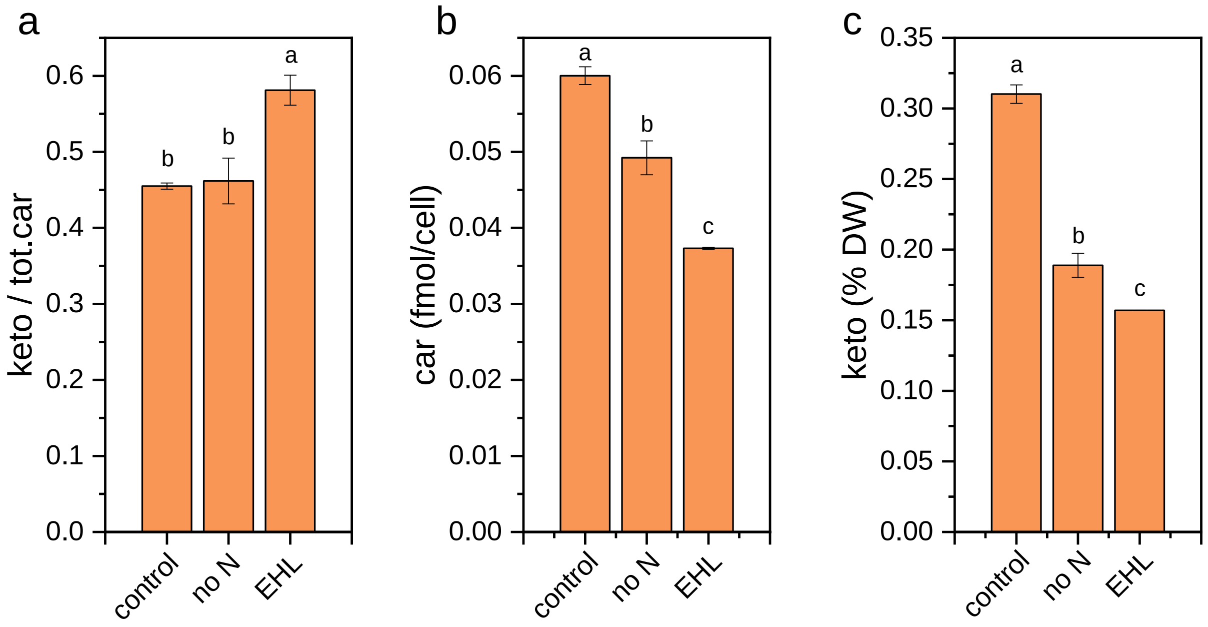

Figure S3. Ketocarotenoid and carotenoid content upon N and high light stress. Cells grown at 150 µmol $m^{-2}s^{-1}$ for 10 days as reported in Figure 5 were centrifuged and resuspended in a medium without nitrogen or exposed at extreme high light (3000 µmol $m^{-2}s^{-1}$) for two days then pigment composition was analysed. Data are expressed as means ± standard deviation (n=3). Significantly different values are indicated with different letters.

Table S1. Pigment analysis of *bkt* lines compared to *LP* background. Raw data for chlorophyll (chl) and carotenoid (car) content per cell and chl/car ratio reported in the main text for *LP* and *bkt* strains (Table 1). *bkt2, bkt3, bktA2* and *bktA5* are different transformed lines expressing CrBKT enzyme.

	chl a (fmol/cell)	car (fmol/cell)	chl/car
LP.1	0.093	0.049	1.909
LP.2	0.097	0.051	1.903
bkt2.1	0.093	0.074	1.255
bkt2.2	0.095	0.075	1.257
bkt3.1	0.103	0.079	1.295
bkt3.2	0.102	0.078	1.299
bktA2.1	0.091	0.071	1.276
bktA2.2	0.088	0.069	1.274
bktA5.1	0.095	0.077	1.227
bktA5.2	0.097	0.079	1.223

Table S2. Carotenoids distribution in *bkt* lines compared to LP background. Raw data for different carotenoid content on total carotenoids reported in the main text for *LP* and *bkt* strains (Table 1). *bkt2, bkt3, bktA2* and *bktA5* are different transformed lines expressing CrBKT enzyme. Carotenoid content is expressed as carotenoid on total carotenoids (mol/mol). viola: violaxanthin; vau: vaucheriaxanthin; antera: anteraxanthin; zea: zeaxanthin; β-car: β-carotene; asta: astaxanthin; adonir: adonirubin; adonix: adonixanthin; cantha: canthaxanthin; keto: ketocarotenoids.

	carotenoids / total carotenoids (mol/mol)									
	viola	asta	vau	antera	zea	cantha	adonir	adonix	β car	keto
LP.1	0.551	0.054	0.184	0.057	0.051	0.031	0.000	0.000	0.072	0.085
LP.2	0.591	0.036	0.184	0.057	0.044	0.022	0.000	0.000	0.066	0.058
bkt2	0.286	0.080	0.131	0.057	0.000	0.258	0.144	0.000	0.044	0.481
bkt3	0.280	0.089	0.113	0.050	0.000	0.254	0.136	0.023	0.056	0.501

Table S3 Chlorophyll and carotenoid content at different light intensities. Pigment content was determined in cells grown in air-lifted photobioreactors at different light intensities (150, 600, 1200 µmol m-2s-1) in ASW medium for 7-11 days . Chlorophyll and carotenoids per cell are expressed as femtomole (fmol). Chl a: chlorophyll a; Car: carotenoids.

	Chl a (fmol/cell)	Car (fmol/cell)
LP 150.1	0.079	0.044
LP 150.2	0.081	0.046
LP 150.3	0.046	0.031
LP 150.4	0.067	0.040
LP 600.1	0.020	0.021
LP 600.2	0.021	0.022
LP 600.3	0.008	0.012
LP 600.4	0.010	0.014
LP 1200.1	0.012	0.016
LP 1200.2	0.012	0.017
LP 1200.3	0.008	0.012
LP 1200.4	0.008	0.012
bkt2 150.1	0.060	0.046
bkt2 150.2	0.046	0.042
bkt2 150.3	0.113	0.069
bkt2 150.4	0.112	0.070
bkt3 150.5	0.101	0.066
bkt3 150.6	0.098	0.064
bkt2 600.1	0.015	0.022
bkt2 600.2	0.015	0.023
bkt2 600.3	0.027	0.026
bkt2 600.4	0.028	0.027
bkt3 600.5	0.028	0.029
bkt3 600.6	0.028	0.030
bkt2 1200.1	0.001	0.010
bkt2 1200.2	0.001	0.010
bkt2 1200.3	0.013	0.016
bkt2 1200.4	0.014	0.017
bkt3 1200.5	0.004	0.014

bkt3 1200.6	0.005	0.015

Table S4 Carotenoid distribution at different light intensities. Pigment content was determined in cells grown in air-lifted photobioreactors at different light intensities (150, 600, 1200 µmol m-2s-1) in ASW medium for 7-11 days . Carotenoids are expressed as carotenoid on total carotenoids (mol/mol). Car: carotenoids; viola: violaxanthin; vaucheria: vaucheriaxanthin; antera: anteraxanthin; zea: zeaxanthin; β-car: β-carotene; asta: astaxanthin; adonir: adonirubin; adonix: adonixanthin; cantha: canthaxanthin; keto: ketocarotenoids.

	carotenoids/total carotenoids (mol/mol)									
	viola	vau	antera	zea	b car	asta	adonir	adonix	cantha	keto
LP 150.1	0.535	0.273	0.048	0.030	0.059	0.037	0.000	0.000	0.018	0.055
LP 150.2	0.525	0.252	0.040	0.031	0.061	0.053	0.000	0.000	0.037	0.090
LP 600.1	0.441	0.200	0.070	0.065	0.054	0.122	0.000	0.000	0.048	0.170
LP 600.2	0.389	0.162	0.072	0.064	0.058	0.177	0.000	0.000	0.077	0.254
LP 1200.1	0.371	0.150	0.075	0.089	0.068	0.166	0.000	0.000	0.082	0.248
LP 1200.2	0.401	0.153	0.054	0.070	0.047	0.182	0.000	0.000	0.093	0.275
bkt 150.1	0.303	0.145	0.040	0.000	0.039	0.083	0.058	0.045	0.286	0.473
bkt 150.2	0.310	0.164	0.040	0.000	0.035	0.091	0.067	0.066	0.227	0.451
bkt 600.1	0.355	0.162	0.042	0.000	0.022	0.078	0.060	0.047	0.234	0.419
bkt 600.2	0.262	0.134	0.041	0.000	0.026	0.085	0.081	0.075	0.296	0.537
bkt 1200.1	0.283	0.203	0.101	0.000	0.008	0.150	0.042	0.022	0.192	0.405
bkt 1200.2	0.366	0.142	0.059	0.000	0.012	0.118	0.042	0.046	0.216	0.421
